# Association Between Serum Uric Acid and Intracranial Arterial Stenosis in a Korean Population: A Secondary Analysis Based on a Cross-Sectional Study

**DOI:** 10.3389/fneur.2022.791456

**Published:** 2022-03-11

**Authors:** Taotao Yao, Anqi Di, Jialing Li, Shuchen Zhang, Jun He, Nuo Xu, Danghan Xu

**Affiliations:** ^1^Rehabilitation Centre, The First Affiliated Hospital of Guangzhou University of Chinese Medicine, Guangzhou University of Chinese Medicine, Guangzhou, China; ^2^Department of Ultrasound, Yancheng First Hospital, Affiliated Hospital of Nanjing University Medical School, Yancheng, China; ^3^Department of Epidemiology, University of Alabama at Birmingham, Birmingham, AL, United States

**Keywords:** uric acid, intracranial arterial stenosis, association, cross-sectional study, Korean population

## Abstract

**Background and purpose:**

Intracranial arterial stenosis (ICAS) is a common cause of cerebrovascular disease. Studies have shown that the disease may be associated with elevated serum uric acid. However, the results remain inexact and controversial. To provide theoretical support for clinical practice, we assessed the relationship between uric acid and ICAS based on previous literature.

**Materials and Methods:**

A total of 1,011 samples were included in the secondary cross-sectional study we investigated. We evaluated the relationship between uric acid level and ICAS using multivariable logistic regression analysis.

**Results:**

The mean age of patients was 64.16 ± 9.13 years, and 35.51% (*n* = 359) were male in the study. One hundred and one (10%) of the included participants had ICAS. In the unadjusted model, uric acid level was positively associated with ICAS [odds ratio (OR) = 1.23, 95% confidence interval (CI): 1.07–1.42, *p* < 0.01]. After adjusting for potential confounders (sex, age, diabetes mellitus, coronary artery occlusive disease, hyperlipidemia, statin medication, hypertension, and fasting glucose), a positive relationship was observed between uric acid and ICAS (OR = 1.26, 95% CI: 1.08–1.47, *p* < 0.05).

**Conclusion:**

There was a positive relationship between uric acid levels and ICAS in neurologically healthy Korean participants.

## Introduction

Intracranial arterial stenosis (ICAS) is an arterial disease associated with inflammatory processes in the intima of arteries and lipid infiltration (1). ICAS is a common cause of cerebrovascular diseases and is more prevalent in Asian and African countries, along with Spain (2, 3). Although ICAS is commonly associated with hypertension, diabetes, hyperlipidemia, and smoking, its relationship with hyperuricemia is rarely mentioned (4). There are conflicting views on the relationship between uric acid and blood vessels. Previous studies have demonstrated that uric acid can act as an antioxidant to prevent atherosclerosis (5). However, recent studies have shown that uric acid levels may be predictive of cerebrovascular disease (6, 7). Intracellular uric acid can result in the production of reactive oxygen species and affect a variety of intracellular signaling pathways. These changes may lead to the development of atherosclerotic lesions (8). In addition, these studies all focused on the possible relationship between uric acid and cerebrovascular diseases, but few have directly proven the relationship between the two. Ahn concluded in a 2018 study of middle-aged South Koreans that serum uric acid was positively correlated with ICAS in women but not in men (9). Conversely, Li et al. found a *U*-shaped relationship between ICAS and serum uric acid levels after adjusting for confounding factors (10). Among the previously published literature, few studies have discussed the relationship between uric acid and ICAS. However, these results remained imprecise and controversial. To provide theoretical support for clinical practice, we conducted a secondary data analysis to investigate the association between uric acid and ICAS based on the open access data from a previously published paper (11), including patients with healthy nervous systems and who may have potential cardiovascular risk factors or a family history of multiple strokes.

## Methods

### Data Sources

As a secondary study, all data in this study were derived from an article published in PLoS One, an open access scientific journal, named “Association between Serum Alkaline Phosphatase Level and Cerebral Small Vessel Disease” (11). The source for research for this study was approved by the Institutional Review Board (IRB) of CHA Bundang Medical Center (IRB No. BD-2010-083). This study did not need to be reviewed by a local ethics committee because it was a secondary analysis. The authors of the original article extracted data from a database from 2008 to 2014. Per the inclusion criteria, 1,441 people were included, and 430 who did not meet the criteria were excluded. The inclusion criteria were as follows: (1) healthy individuals with underlying cardiovascular risk factors or who had multiple strokes in their family, (2) aged ≥ 45 years, (3) patients who have undergone magnetic resonance imaging (MRI) and magnetic resonance angiography (MRA) scans of the brain. Moreover, the exclusion criteria were as follows: (1) inadequate medical information, (2) no laboratory tests performed, (3) no data on brain MRI or MRA assessments, (4) previous history of neurological disease, and (5) abnormal neurological findings at the time of examination.

### Measurement of Variables

The variables included were age, smoking, sex, systolic blood pressure (SBP), diabetes mellitus (DM), diastolic blood pressure (DBP), hyperlipidemia, coronary artery occlusive disease (CAOD), hypertension, statin medication, white blood cell (WBC) count, hematocrit, platelet count, estimated glomerular filtration rate (eGFR), glutamic oxaloacetic transaminase (GOT), fasting glucose, glutamic pyruvic transaminase (GPT), total cholesterol, alkaline phosphatase, triglyceride, uric acid, moderate-to-severe cerebral white matter hyperintensities (MS-cWMH), silent lacunar infarct (SLI), extracranial arterial stenosis (ECAS), and large cerebral arterial stenosis (LCAS). Hypertension refers to repeated measurements ≥140 of SBP or DBP of ≥90 mmHg or taking antihypertensive drugs. Patients with DM are referred to those who regularly take diabetes medication or have fasting glucose > 126 mg/dl. Smoking refers to either smoking in the current year or the year prior. If the patient had acute myocardial infarction or unstable angina pectoris and was diagnosed with CAOD by an auxiliary assessment, CAOD was considered. SLI was a small cavitated lesion that deeply penetrates the arterial supply zone (12). Uric acid was categorized into three groups: first tertile < 3.7 mg/dl; second tertile 3.7–5.0 mg/dl; third tertile ≥5.0 mg/dl. LCAS was identified as significant intracranial or extracranial cerebral artery stenosis (≥50%) or complete occlusion on cerebral MRA (13). ICAS can occur in a variety of cerebral arteries, such as the anterior cerebral, posterior cerebral, middle cerebral, distal vertebral, distal internal carotid, and basilar arteries. Other data were obtained *via* laboratory experiments, wherein eGFR was calculated using the abbreviated Modification of Diet in Renal Disease Study Equation (14).

### Statistical Analysis

Continuous variables were described as mean ± standard deviation (normal distribution) or median (skewed distribution). Categorical variables were presented as percentages and frequencies. We used a one-way analysis of variance (ANOVA; for normal distribution), the Kruskal-Wallis H (for skewed distribution) tests, and chi-square tests (categorical variables) to obtain any statistical differences across the ICAS and non-ICAS groups. To assess the relationship between uric acid levels and ICAS, we conducted a univariate logistic regression analysis. Adjusted confounders were chosen based on *p*-values and clinical rationale. We presented non-adjusted and multivariable-adjusted models. We simultaneously showed three results, namely, the unadjusted, minimally adjusted (sex, age), and fully adjusted analysis results (sex, age, diabetes mellitus, CAOD, hyperlipidemia, statin medication, hypertension, and fasting glucose). Furthermore, we also performed a generalized additive model (GAM) to identify any non-linear relationships. The maximum model likelihood was used when the ratio between ICAS and uric acid showed a significant change in the smoothing curve (15). Stratified logistic regression models were used for subgroup analyses, and the modification and interaction of subgroups were detected using the likelihood ratio test. We used R (R Foundation; http://www.R-project.org; version 3.4.3) and EmpowerStats (www.empowerstats.com; version 2.20; X&Y Solutions, Inc., Boston, MA) to perform data analysis.

## Results

### Baseline Characteristics of Participants

One hundred and one (10%) participants had ICAS. The mean age was 64.16 ± 9.13 years, and 35.51% (*n* = 359) of patients were male. There were no statistically significant differences in smoking, sex, DBP, hyperlipidemia, statin medication, hematocrit, platelet count, GPT, total cholesterol, ALP, or GOT among the different uric acid groups (*p* > 0.05). However, there were significant differences in age. Particularly, the non-ICAS group (63.7 ± 9.1 years) was significantly younger than those in the ICAS group (68.1 ± 8.8 years). Regarding hypertension, fewer patients had hypertension in the non-ICAS group (55.8%) than in the ICAS group (70.3%). Results were similar for DM, in that patients with ICAS (36.6%) were more likely to have DM than those who did not (20.6%). The proportion of patients with MS-cWMH, SLI, LCAS, and ECAS was much higher in the ICAS group. Other included variables, such as SBP, CAOD, WBC, eGFR, fasting glucose, MS-cWMH, triglyceride, and uric acid, were also statistically significant (*p* < 0.05). The patients' baseline characteristics are presented in Table 1.

**Table 1:** Baseline characteristics of study population by ICAS.

**Characteristics**	**With ICAS**		***p*-value**
	**No**	**Yes**	
	**(*n* = 910)**	**(*n* = 101)**	
Age (years)	63.7 ± 9.1	68.1 ± 8.8	**<** **0.001**
Sex (%)			0.803
Male	322 (35.4)	37 (36.6)	
Female	588 (64.6)	64 (63.4)	
Smoking (%)	188 (20.7)	17 (16.8)	0.364
SBP (mmHg)	130.9 ± 17.8	138.0 ± 18.0	**<** **0.001**
DBP (mmHg)	79.9 ± 11.5	81.4 ± 12.2	0.229
Hypertension (%)	508 (55.8)	71 (70.3)	**0.005**
Diabetes mellitus (%)	187 (20.6)	37 (36.6)	**<** **0.001**
Hyperlipidaemia (%)	296 (32.5)	36 (35.6)	0.527
CAOD (%)	42 (4.6)	10 (9.9)	**0.023**
Statin medication (%)	197 (21.7)	30 (29.7)	0.066
WBC ( ×10^9^/L)	6.5 ± 1.9	7.0 ± 2.4	**0.013**
Hematocrit (%)	40.1 ± 3.8	39.8 ± 5.2	0.527
Platelet ( ×10^9^/L)	232.0 ± 54.0	231.1 ± 54.6	0.867
eGFR (mL/min/1.73 m^2^)	74.8 ± 16.6	68.3 ± 18.0	**<** **0.001**
Fasting glucose (mg/dL)	125.6 ± 44.9	140.5 ± 52.2	**0.002**
GOT (IU/L)	23.2 ± 7.9	23.7 ± 8.5	0.529
GPT (IU/L)	23.1 ± 12.5	23.7 ± 13.0	0.660
Total cholesterol (mg/dL)	193.7 ± 38.6	193.3 ± 45.9	0.913
Triglyceride (mg/dL)	145.2 ± 85.7	164.4 ± 96.8	**0.037**
ALP (IU/L)	181.7 ± 55.3	187.9 ± 57.5	0.291
Uric acid (mg/dL)	4.5 ± 1.4	4.9 ± 1.5	**0.003**
Uric acid (mg/dL) tertiles			0.069
<3.7 mg/dL	279 (30.9)	23 (22.8)	
3.7–5.0 mg/dL	328 (36.4)	34 (33.7)	
≥5.0 mg/dL	295 (32.7)	44 (43.5)	
MS-cWMH (%)	251 (27.6)	43 (42.6)	**0.002**
SLI (%)	89 (9.8)	31 (30.7)	**<** **0.001**
LCAS (%)	92 (10.1)	101 (100.0)	**<** **0.001**
ECAS (%)	93 (10.2)	25 (24.8)	**<** **0.001**

*The independent t-tests (for continuous variables) and the Chi-squared tests (for categorical variables) were performed to identify differences by ICAS*.

*SBP, systolic blood pressure; DBP, diastolic blood pressure; CAOD, coronary artery occlusive disease; WBC, white blood cells; eGFR, estimated glomerular filtration rate; GOT, glutamic oxaloacetic transaminase; GPT, glutamic pyruvic transaminase; ALP, alkaline phosphatase; MS-cWMH, moderate-to-severe cerebral white matter hyperintensities; SLI, silent lacunar infarct; LCAS, large cerebral arterial stenosis; ECAS, extracranial arterial stenosis*.

*The data in bold are statistically significant*.

### Univariate Analysis

Table 2 shows the results of univariate analysis. Age, SBP, hypertension, white blood cell count, diabetes mellitus, CAOD, uric acid estimated glomerular filtration rate, and fasting glucose were associated with ICAS. The odds ratios [ORs; 95% confidence intervals (CIs)] of eGFR and uric acid were.98 (95% CI: 0.96,0.99) and 1.23 (95% CI: 1.07, 1.42), respectively, indicating that eGFR is inversely associated with ICAS, while ICAS is positively associated with uric acid. Smoking, DBP, hyperlipidemia, statin medication, hematocrit, platelet, GOT, GPT, total cholesterol, triglyceride, and alkaline phosphatase were not associated with ICAS.

**Table 2:** Univariate analysis of ICAS.

**Variables**	**ICAS**	
	**OR (95% CI)**	***p*-value**
Age (years)	1.06 (1.03, 1.08)	**<0.0001**
Sex		
Male	Ref.	
Female	0.95 (0.62, 1.45)	0.8035
Smoking	0.78 (0.45, 1.34)	0.3651
SBP (mmHg)	1.02 (1.01, 1.03)	**0.0002**
DBP (mmHg)	1.01 (0.99, 1.03)	0.2291
Hypertension	1.87 (1.20, 2.93)	**0.0059**
Diabetes mellitus	2.24 (1.45, 3.46)	**0.0003**
Hyperlipidaemia	1.15 (0.75, 1.77)	0.5272
CAOD	2.27 (1.10, 4.68)	**0.0261**
Statin medication	1.53 (0.97, 2.41)	0.0673
WBC ( ×10^9^/L)	1.13 (1.03, 1.25)	**0.0132**
Hematocrit (%)	0.98 (0.93, 1.04)	0.5267
Platelet ( ×10^9^/L)	1.00 (1.00, 1.00)	0.8666
eGFR (mL/min/1.73 m^2^)	0.98 (0.96, 0.99)	**0.0003**
Fasting glucose (mg/dL)	1.01 (1.00, 1.01)	**0.0023**
GOT (IU/L)	1.01 (0.98, 1.03)	0.5290
GPT (IU/L)	1.00 (0.99, 1.02)	0.6602
Total cholesterol (mg/dL)	1.00 (0.99, 1.00)	0.9133
Triglyceride	1.00 (1.00, 1.00)	0.0388
ALP (IU/L)	1.00 (1.00, 1.01)	0.2913
Uric acid (mg/dL)	1.23 (1.07, 1.42)	**0.0033**
Uric acid (mg/dL) tertiles		
1st tertile (<3.7 mg/dL)	Ref.	
2nd tertile (3.7–5.0 mg/dL)	1.26 (0.72, 2.19)	0.4166
3rd tertile (≥5.0 mg/dL)	1.81 (1.06, 3.07)	**0.0284**

*Logistic regression was performed for univariate analysis*.

*ICAS, intracranial arterial stenosis; OR, odds ratio; CI, confidence interval; Ref, reference group; SBP, systolic blood pressure; DBP, diastolic blood pressure; CAOD, coronary artery occlusive disease; WBC, white blood cells; eGFR, estimated glomerular filtration rate; GOT, glutamic oxaloacetic transaminase; GPT, glutamic pyruvic transaminase; ALP, alkaline phosphatase; MS-cWMH, moderate-to-severe cerebral white matter hyperintensities; SLI, silent lacunar infarct*.

*The data in bold are statistically significant*.

### Subgroup Analysis Results

**Figure 1:**
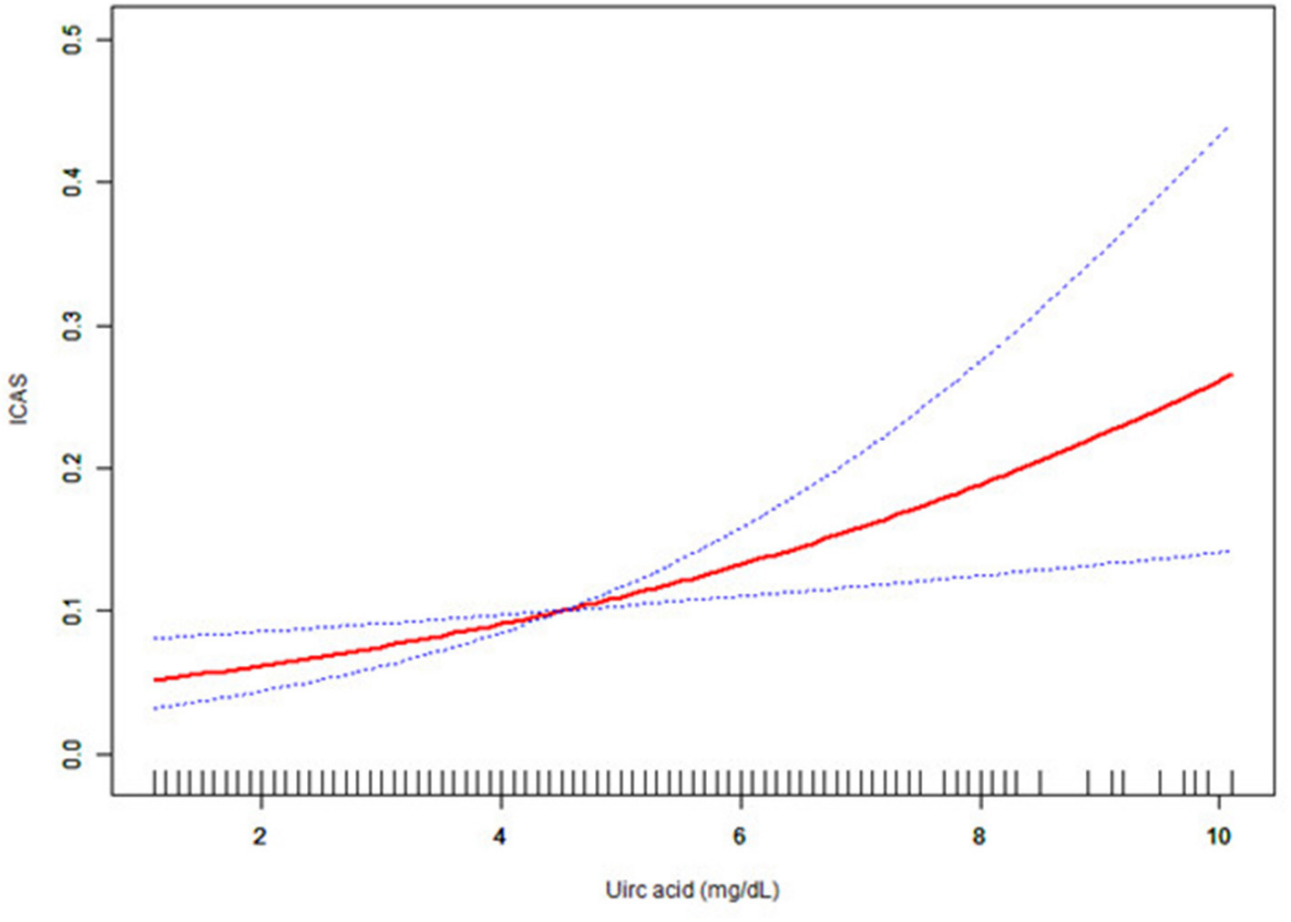
The effect of uric acid on intracranial arterial stenosis (ICAS) in subgroup size forest plot. CAOD, coronary artery occlusive disease.

The interactions were not statistically significant for age, smoking, sex, hypertension, diabetes mellitus, hyperlipidemia, CAOD, and statin medication (*p* > 0.05), as shown in Figure 1. In summary, there was no evidence of any difference between uric acid and ICAS in the subgroup analyses.

### The Results of the Relationship Between Uric Acid and ICAS

Multivariable logistic regression models were used to assess the association between uric acid and ICAS. In addition, the unadjusted, adjusted I (sex, age), and adjusted II (sex, age, diabetes mellitus, CAOD, hyperlipidemia, statin medication, hypertension, and fasting glucose) models are shown in Table 3. In the unadjusted model, uric acid level was positively associated with ICAS (OR = 1.23, 95% CI: 1.07–1.42, *p* < 0.01). In the adjusted I (sex, age) model, the results showed a positive association with ICAS (OR = 1.25, 95% CI: 1.08–1.45, *p* < 0.01). After adjusting for potential confounders (sex, age, diabetes mellitus, CAOD, hyperlipidemia, statin medication, hypertension, and fasting glucose), a positive relationship was revealed between uric acid and ICAS (OR = 1.26, 95% CI: 1.08–1.47, *p* < 0.05). The uric acid values were divided into three sections with 3.7 mg/dl and 5.1 mg/dl as the boundaries for each analysis. After adjusting for various confounders, results showed that compared with the lowest tertile group (first tertile) the adjusted OR (95% CI) of ICAS was 1.31 (0.74, 2.33) in the second and 1.98 (1.12, 3.48) in the third tertile.

**Table 3:** Multivariable logistic regression analysis of uric acid in the presence of ICAS.

**Logistic**	**ICAS**					
**regression**	**Unadjusted**		**Adjust I**		**Adjusted II**	
**model**	**OR (95% CI)**	***p*-value**	**OR (95% CI)**	***p*-value**	**OR (95% CI)**	***p*-value**
Uric acid (mg/dL)	1.23 (1.07, 1.42)	0.0033	1.25 (1.08, 1.45)	0.0035	1.26 (1.08, 1.47)	0.0033
Uric acid (mg/dL) (tertile)						
1st tertile(<3.7 mg/dL)	Ref.		Ref.		Ref.	
2nd tertile(3.7-5.0 mg/dL)	1.26 (0.72, 2.19)	0.4166	1.31 (0.75, 2.30)	0.3440	1.31 (0.74, 2.33)	0.3512
3rd tertile(≥5.0 mg/dL)	1.81 (1.06, 3.07)	0.0284	1.87 (1.07, 3.27)	0.0276	1.98 (1.12, 3.48)	0.0180

*Logistic regression was performed for subgroup analysis*.

Adjust I model adjust for: sex, age (years).

Adjust II model adjust for: sex, age (years), hypertension, diabetes mellitus, hyperlipidaemia, CAOD, statin medication and fasting glucose (gm/dL).

*ICAS, intracranial arterial stenosis; Ref, reference group; OR, odds ratio; CI, confidence interval; CAOD, coronary artery occlusive disease*.

### Curve Fitting Analyses

**Figure 2:**
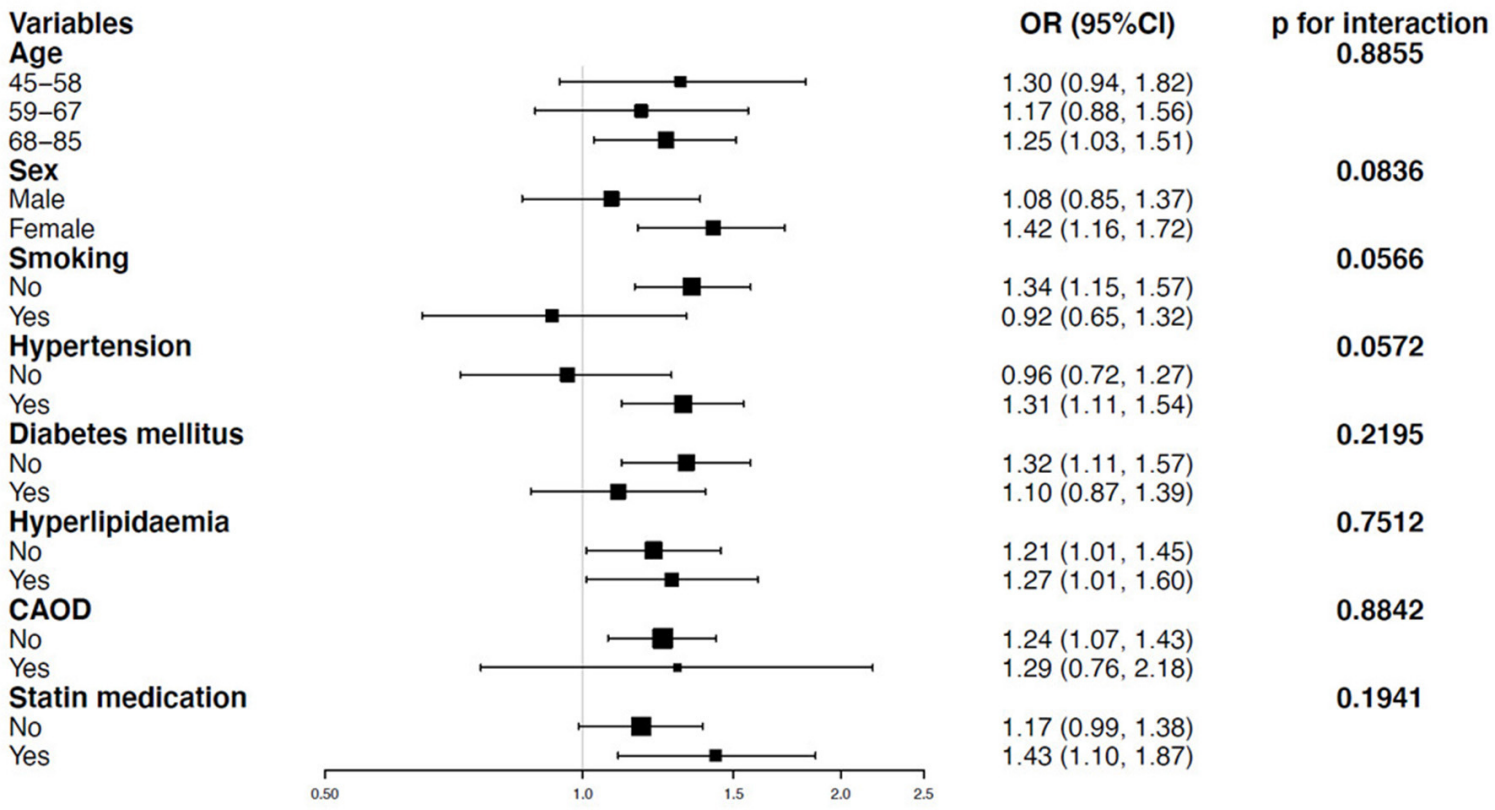
Association between uric acid level and the presence of ICAS. The solid red and dashed blue lines reflect the estimated probability and 95% CI for the presence of ICAS, respectively, and were drawn using the generalized additive model. Adjusted for sex, age (years), hypertension, diabetes mellitus, hyperlipidemia, coronary artery occlusive disease (CAOD), statin medication, and fasting glucose level (g/dl). ICAS, intracranial arterial stenosis; CAOD, coronary artery occlusive.

As uric acid was a continuous variable, curve-fitting analysis was required. In this study, we found a linear relationship between uric acid and ICAS (Figure 2).

## Discussion

Uric acid is a product of purine metabolism that occurs in the liver (16). Its antioxidant properties play a dual role in the protection and promotion of oxidation formation. For example, high uric acid levels may lead to gout and are also associated with hypertension, atherosclerosis, insulin resistance, and diabetes (17). Previous studies have suggested that uric acid and cardiovascular diseases are closely related. However, few studies have focused on cerebrovascular diseases (18). In a 2020 study (19), Talebi concluded that the increased serum uric acid may be associated with ischemic stroke, which warrants further investigation, but certainly implies the potential other effects of uric acid. Studies have shown that large amounts of uric acid crystals are present in atherosclerotic plaques (20). This may be due to lipid peroxidation which creates oxy-radicals for uric acid. This leads to inflammation of the vascular wall, which is exacerbated by the accumulation of uric acid crystals and promotes atherosclerosis (21, 22).

Therefore, in this study, a cross-sectional analysis was used to identify the relationship between uric acid and ICAS. As observed in this cross-sectional secondary analysis, with a higher uric acid level, the odds of having ICAS are also higher. Therefore, the two are positively correlated. This is consistent with the results of previous studies. According to an article published by Song in 2018 (23), the cumulative incidence of vascular stenosis is proportional to the amount of uric acid.

To assess the relationship between uric acid level and ICAS, we conducted a univariate analysis. In the univariate analysis, uric acid was positively associated with ICAS (OR = 1.23; 95% CI: 1.07–1.42, *p* = 0.0033). Moreover, age, SBP, CAOD, hypertension, eGFR, diabetes mellitus, WBC count, and fasting glucose level also showed significant relationships with ICAS.

We also conducted subgroup analyses and tested for *p*-values for interactions. Among all subgroups that we tested, we failed to find any group that had a *p*-value for interaction under.05. As shown in Figure 1, there was a positive association between uric acid level and ICAS in the 68 to 85-year-old age group (OR = 1.25, 95% CI: 1.03–1.51, *p* = 0.0208). We found that the relationship between uric acid and ICAS was significant only in the female population (OR = 1.42, 95% CI: 1.16–1.72, *p* = 0.0005), which was consistent with the results obtained by Ahn et al. (9). In fact, the level and rate of increase in serum uric acid are closely related to age and sex. In addition, hyperuricemia is much more common in women over 65 years of age than in men, which may explain our results (24). There was a positive association between smoking and sex (OR = 1.34, 95% CI: 1.15–1.57, *p* = 0.0003). However, this is likely because we included a majority of women in our included data, and women smoked at a lower rate. In addition, it may be because of the cross-sectional nature of the study that this group of non-smokers may already have had health problems and, therefore, do not smoke. There was also a positive relationship between uric acid level and ICAS in patients with hypertension (OR = 1.31, 95% CI: 1.11–1.54, *p* = 0.0013). Hypertension is a risk factor for cerebrovascular events, which is consistent with the situation observed in clinical practice (25). Hyperlipidemia is another clinical risk factor (26). Clinically, statins are often used to reduce blood lipid levels to protect the heart and brain vessels (27). However, our data revealed a positive association between uric acid and ICAS, regardless of whether patients had hyperlipidemia or were taking statins (all OR > 1.00, *p* < 0.05). This suggests very stable relationship between uric acid and ICAS.

To test the independent impact of uric acid on ICAS, we used multivariable logistic regression models. In the adjusted I model, after adjusting for sex and age, we found a positive relationship between uric acid level and ICAS (OR = 1.25, 95% CI: 1.08–1.45, *p* = 0.0035). When we further considered sex, hypertension, age, diabetes mellitus, hyperlipidemia, CAOD, fasting glucose, and statin medication, the relationship between uric acid and ICAS remained unchanged (OR = 1.26, 95% CI: 1.08–1.47, *p* = 0.0033) in the adjusted II model. We also created a figure showing the relationship between uric acid and ICAS with consideration of confounders (Figure 2). Our results differ from those of previous research (10). Following a cross-sectional analysis, our conclusion is that the relationship between uric acid and ICAS is roughly represented by an upward curve, while that suggested by the previous literature is *U*-shaped. We considered that the difference was caused by different study populations, a higher outcome proportion, and the different proportions of the exposure categories from the previous study. In addition, a multivariable logistic regression analysis in another study showed that uric acid was an independent predictor of intracranial stenosis in elderly patients (OR = 1.003, 95% CI: 1.000–1.007, *p* = 0.042) (28). This study provides support to our conclusions.

This study had several limitations. First, since this was a secondary study, extrapolation of the final conclusion requires careful consideration. Further, cross-sectional studies cannot reveal cause and effect. In contrast to other studies exploring uric acid and cerebrovascular diseases (9, 10), we focused more on the relationship between uric acid and ICAS in neurologically healthy participants. Due to the cross-sectional study design, we conducted subgroup analyses and multivariable logistic regression analyses, thereby weakening the influence of confounding factors on the conclusion to the greatest extent. Our results point to the key role of alerting healthy individuals with high uric acid levels to the potential aspects of ICAS.

## Data Availability Statement

The original contributions presented in the study are included in the article/supplementary material, further inquiries can be directed to the corresponding author/s.

## Ethics Statement

The studies involving human participants were reviewed and approved by the Institutional Review Board (IRB) of CHA Bundang Medical Center (IRB No. BD-2010-083). The patients/participants provided their written informed consent to participate in this study.

## Author Contributions

DX proposed the concept of the manuscript and assisted with the statistical analysis and revision of the manuscript. TY and AD contributed to the manuscript drafting. NX contributed to data analysis and interpretation. JL, SZ, and JH contributed to the study conception and revision of the manuscript. All authors have read and approved the final manuscript.

## Conflict of Interest

The authors declare that the research was conducted in the absence of any commercial or financial relationships that could be construed as a potential conflict of interest.

## Publisher's Note

All claims expressed in this article are solely those of the authors and do not necessarily represent those of their affiliated organizations, or those of the publisher, the editors and the reviewers. Any product that may be evaluated in this article, or claim that may be made by its manufacturer, is not guaranteed or endorsed by the publisher.
